# Observation of the efficacy of parathyroidectomy for secondary hyperparathyroidism in hemodialysis patients: a retrospective study

**DOI:** 10.1186/s12893-023-02143-y

**Published:** 2023-08-12

**Authors:** Wenqiang Qiu, Ge Zhou

**Affiliations:** 1 Department of General Surgery, Jinzhou Medical University Postgraduate Training Base (Liaoyang Central Hospital), Liaoyang, 111000 China; 2 Department of General Surgery, Liaoyang Central Hospital, Liaoyang, China

**Keywords:** Secondary hyperparathyroidism, Hemodialysis, Parathyroidectomy, Quality of life

## Abstract

**Purpose:**

Parathyroidectomy (PTX) is commonly performed as a treatment for secondary hyperparathyroidism (SHPT) in patients with end-stage renal disease (ESRD). We aimed to evaluate the efficacy of PTX in patients with SHPT who underwent hemodialysis.

**Methods:**

This retrospective study analyzed the clinical treatment of 80 hemodialysis patients with SHPT who underwent either total PTX with forearm auto transplantation (TPTX + AT) or subtotal parathyroidectomy (SPTX). We compared the changes in biochemical indices before and after surgery as well as the attenuation of intact parathyroid hormone (iPTH) in the TPTX and SPTX groups. We also evaluated clinical symptoms and quality of life using the Visual Analog Scale (VAS) and the Short Form-36 Questionnaire (SF-36) before and at 3, 6, and 12 months after surgery.

**Results:**

Serum iPTH and serum phosphorus levels decreased significantly after surgery in 80 patients with SHPT (*P* < 0.05). Within one month of surgery, there was a difference in iPTH levels between the TPTX + AT and SPTH groups, but there was no difference over time. Patients experienced significant improvement in their clinical symptoms of restless leg syndrome, skin itching, bone pain, and joint pain at 1 week post operation (*P* < 0.001). Quality of life significantly improved after surgery, as assessed by SF-36 scores (*P* < 0.05). Hypocalcemia was the most common postoperative complication, occurring in 35% of patients. Within the first 12 months post surgery, 5 patients had a recurrence.

**Conclusion:**

PTX is effective in rapidly reducing iPTH levels, improving calcium and phosphorus metabolism disorders, and enhancing patients’ quality of life by safely and effectively relieving clinical symptoms.

## Introduction

Chronic kidney disease (CKD) frequently complicates secondary hyperparathyroidism (SHPT), which is characterized by abnormal metabolism of calcium and phosphorus that triggers compensatory hyperplasia of the parathyroid gland and increased secretion of intact parathyroid hormone (iPTH) throughout the gland. Patients suffering from SHPT are prone to various complications, including hyperparathyroidism, hypercalcemia or hypocalcemia, persistent hyperphosphatemia, and disturbances in the functions of the bone (e.g., bone pain, deformities) [1, neurological system (e.g., insomnia) [2, hematological system (e.g., anemia and coagulation dysfunction) [3, and cardiovascular system (e.g., arterial sclerosis) [4. Additionally, patients may experience skin itching, and joint pain which can significantly impair their quality of life. Moreover, long-term chronic electrolyte and biochemical disorders can increase the incidence of cardiovascular events and all-cause mortality [5, 6].

At present, primary treatments for SHPT include medical and surgical interventions, alongside emerging therapies such as photodynamic therapy [7. Although low-phosphate diets, hemodialysis, and drug treatment can to a certain extent reduce iPTH levels in early and middle-stage patients, drug treatment has little effect on improving high iPTH, ion metabolism disorders, and symptoms for refractory or late-stage SHPT patients [8. Since Stanbury first proposed SHPT in 1960, parathyroidectomy (PTX) has been an effective treatment [9. The 2017 clinical practice guidelines for chronic kidney disease-mineral and bone disorder (CKD-MBD) recommend PTX as the preferred therapy for refractory SHPT. This surgical intervention is recommended over medical treatments due to its superior effectiveness in controlling iPTH levels and addressing the underlying complications of SHPT [10. There are three main types of PTX: subtotal parathyroidectomy (SPTX), total parathyroidectomy (TPTX), and total parathyroidectomy with auto transplantation (TPTX + AT) [11, 12]. According to reports, PTX can increase the survival rate of dialysis patients by 15–57% and can improve hypercalcemia, hyperphosphatemia, tissue calcification, bone mineral density, and health-related quality of life (QOL) [13.

QOL is a significant indicator used to assess the health status of SHPT patients. It renders a better evaluation of their disease prognosis and the effectiveness of PTX treatment and has a close association with long-term survival. The Short Form-36 Questionnaire (SF-36) may be a reliable instrument for evaluating quality of life in individuals with SHPT who have undergone parathyroid surgery as well as a powerful predictor of morbidity and unfavorable outcomes in dialysis patients [14, 15]. Inaddition, the Visual Analog Scale (VAS) is the commonly used subjective evaluation method for quantifying sensation or experience, such as pain, emotion, and satisfaction. However, to date, no studies have combined these two scales to offer a more thorough evaluation of the effectiveness of PTX. Herein, this retrospective study meticulously examines and juxtaposes blood biochemical indices, quality of life, and clinical symptoms in hemodialysis patients both pre and post PTX treatment over an extended duration, presenting a comprehensive evaluation of the efficacy of PTX.

## Materials and methods

### Study sign and patients

**Fig. 1:**
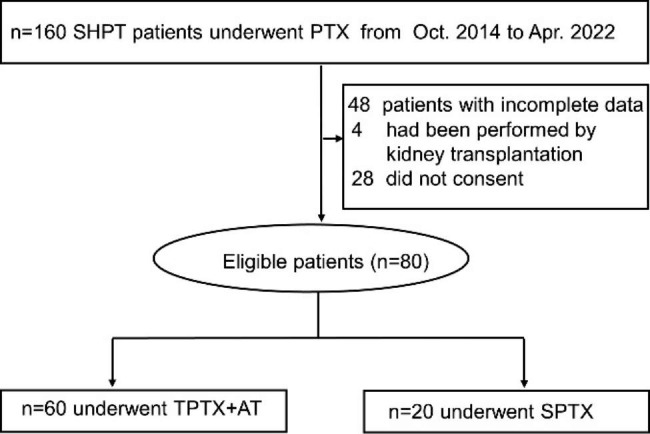
Flowchart of review grouping of the study

This retrospective single-center study reviewed the electronic medical records of 160 uremic patients who underwent long-term hemodialysis and parathyroidectomy (PTX) at our hospital between October 2014 and April 2022. Following the exclusion of 80 individuals for noncompliance, the study also included an additional 80 participants, and 60 patients underwent TPTX + AT surgery, while 20 underwent SPTX surgery. The review and grouping of the study are shown in Fig. 1. The inclusion criteria were as follows: (1) age ≥ 18 years, (2) hemodialysis maintenance at least twice weekly for more than 3 months, and (3) met the diagnostic criteria for refractory secondary hyperparathyroidism (SHPT): serum intact parathyroid hormone (iPTH) > 600–800 ng/L and accompanying high calcium level or hyperphosphatemia, severe bone pain, itchy skin, extraosseous calcification and deformity, failure of medical treatment, and imaging examination identifying at least one enlarged parathyroid gland (4). Successful PTX surgery. Compared with preoperative monitoring, intraoperative monitoring of iPTH resulting in a rate of decline > 70% was the criterion for a successful operation [16. Exclusion criteria included significant cardiovascular disease, active inflammation or infection, malignancy, and treatment with steroids and/or immunosuppressants. The study was approved by the Ethics Committee of the Liaoyang Central Hospital, and all participants provided signed informed consent.

### Surgical methods and perioperative management

All patients underwent a comprehensive preoperative evaluation upon admission to exclude any surgical contraindications. The evaluation included a series of tests such as complete blood cell counts, serum electrolytes, chest X-rays, electrocardiograms, coagulation function screening, and parathyroid ultrasound. TPTX + AT or SPTX are the surgical procedures employed by experienced attending surgeons in our hospital. Hemodialysis is performed within 24 h postoperation using high-calcium dialysate of 1.75 mmol/L. To avoid postoperative bleeding, no heparin dialysis was used for a week after surgery. The primary aim of hemodialysis is to remove toxins in a timely manner and maintain electrolyte stability, and patients receive hemodialysis three times a week at our hospital’s hemodialysis center thereafter. Intravenous calcium gluconate of 8–16 g/24 h is administered within 2–3 weeks postsurgery, with routine administration of calcitriol of 0.25–2.5 ug/d, to maintain the total serum calcium level between 1.8 and 2.2 mmol/L.

### Observation indicators

The evaluated biochemical parameters were the levels of serum intact parathyroid hormone (iPTH, reference range (RR): 15–65 pg/mL), total calcium (Ca, RR: 2.11–2.52 mmol/L), phosphorus (P, RR: 0.85–1.51 mmol/L), hemoglobin (Hb, RR: 130–175 g/L) alkaline phosphatase (ALP, RR: 45–125 U/L). All laboratory tests were performed by highly skilled laboratory physicians, who followed standardized procedures.

The health status of patients was evaluated using the Chinese version of the medical outcomes study 36-item short from health survey (SF-36), which is a widely validated and commonly used generic tool belonging to the international quality of life assessment program. It is easy to use, acceptable to patients, and fulfils stringent criteria of reliability and validity [17. The SF-36 has been translated and applied in over 50 countries, and it measures eight domains, including physical function (PF), role-functioning physical (RP), bodily pain (BP), general health (GH), vitality (VT), social functioning (SF), role-functioning emotional (RE), and mental health (MH), with scores ranging from 0 to 100. The physical health summary score (PCS) and the mental health summary score (MCS) were also calculated. Higher scores indicate better self-perceived health and a higher quality of life, while a change of at least 2 points in the summary scores is a clinically significant indicator of changes in health status. Furthermore, the Visual Analog Scale (VAS) was employed to appraise the primary symptoms of restless legs syndrome, skin itching, bone pain, joint pain, and insomnia, with each item on a score range of 0–10. Higher VAS scores indicated more severe symptoms. All aforementioned scores were assessed by specialized physicians during patient hospitalization, outpatient dialysis, or follow-up visits.

If the total serum calcium concentration was lower than 1.875 mmol/L 72 h after surgery, it was defined as severe postoperative hypocalcemia. The corrected value of serum albumin was calculated as follows : corrected value (mmol/L) = total calcium concentration + 0.8 × [40 - serum albumin]. Postoperative hyperkalemia was defined as a postoperative serum potassium concentration exceeding 5.5 mmol/L. Recurrent secondary hyperparathyroidism (SHPT) is diagnosed if iPTH ≥ 300 pg/mL or 9 times higher than the upper limit of normal and symptoms such as bone pain and itching appear 6 months postsurgery. If the iPTH level was below 10 pg/mL after 1 year of follow-up, hypoparathyroidism was defined. Graft survival should be evaluated based on the normalization of blood iPTH levels within three months.

### Statistical analysis

Statistical analysis was conducted on a sample of n = 80 patients using SPSS version 26 for Windows. The mean (standard deviation) and median (interquartile range) were used to report continuous variables with normal and nonnormal distribution respectively. Paired t-tests and nonparametric tests (Wilcoxon signed-rank test) were used to compare continuous variable data. To compare the variations in metrics between the TPTX + AT and SHPT groups, we employed the Student’s t-test or the Mann-Whitney U test. The chi-square test was employed to compare postoperative complication rates. Statistical significance was defined at *p* < 0.05.

## Results

### Case report on parathyroid gland

The pathological analysis of 320 excised parathyroid glands from 80 patients revealed the presence of parathyroid adenoma (4 cases) and parathyroid nodular hyperplasia (316 cases). Among our patients, 2 underwent removal of 2 glands, 3 had 3 glands removed, 69 had all 4 glands removed,5 had 5 glands removed, and 1 had all six glands removed. Notably, further imaging or laboratory studies confirmed residual parathyroid glands in patients who had less than complete gland removal.

### Preoperative demographic and laboratory findings

The 80 patients who underwent surgery before April 2022 are summarized in Table 1. Of these patients, 45% (36) were female, with a mean (SD) age of 46.32 (11.81) years and a mean dialysis duration of 8.55 (4.22) years. The most common primary disease was chronic glomerulonephritis, accounting for approximately 38.75% [31] of cases, followed by diabetic nephropathy, accounting for approximately 22.5% [18] of cases.

**Table 1:** Patient characteristics and baseline data

Characteristics	Rang or n (%)
Female	36(45%)
Age(years)	46.32 ± 11.81
Dialysis vintage (years)	8.55 ± 4.22
Primary disease	
Chronic glomerulonephritis	31(38.75%)
Diabetic nephropathy	18(22.5%)
Hypertensive nephropathy	14(17.5)
Obstructive nephropathy	7(8.76%)
Others	10(12.5%)
Albumin(g/L)	41.24 ± 3.82
Creatinine (µmol/L)	865.34(674.00 ~ 1108.15)

Data are presented as n (%), and continuous data are presented as the mean ± SD when normally distributed and as median (interquartile range) when skewed; Normal ranges: Albumin (40-55 g/L); Creatinine (57-97µmol/L)

### Comparison of blood biochemical indicators before and after surgery

As summarized in Table 2, there was no significant difference in hemoglobin levels between preoperative and postoperative measurements at different time points. Alkaline phosphatase (ALP) levels increased significantly in the first week after surgery compared to preoperative levels(*P* < 0.05) but did not show a significant difference from preoperative levels at 1 month postoperatively. By approximately 3 months after surgery, the median ALP levels had returned to normal levels. Immediately after the surgery, all participants observed a substantial reduction in their levels of iPTH, serum calcium, and phosphorus when compared to their corresponding preoperative levels. On the third day after the operation, significant reduction in the serum calcium levels was observed, with mean value of 2.09 ± 0.25mmol/L (Table 3).

**Table 2:** Comparison of hemoglobin and alkaline phosphatase before and after surgery

	Hemoglobin(g/L)	Alkaline phosphatase(U/L)
Pre-operation	111.7 ± 21.81	234.8(130.3,427.3)
1 wk	112.01 ± 16.26	274.8(156.28,452.98) *
1 mo	109.48 ± 17.12	207.5(135,414.25)
3 mo	113.36 ± 21.06	91.9(68.33,133.15) *
6 mo	109.66 ± 15.67	85(65,125) *
12 mo	110.64 ± 19.84	82(66.5,112)*

Date are expressed as the mean ± SD or median (interquartile range). A probability value of *p < 0.05 was considered to be statistically significant compared with those before the operation; wk, week; mo, month;

**Table 3:** Comparison of iPTH, serum calcium and phosphorus levels before and after PTX

	iPTH(pg/ml)	Serum calcium(mmol/L)	Serum phosphorus(mmol/L)
Pre-operation	1239.42(1621.8,2322.26)	2.49 ± 0.23	2.28 ± 0.54
5 min	207.36(303.35,454.62)*	/	/
10 min	149.27(219.39,289.9)*	/	/
30 min	111.65(148.4,242.86)*	/	/
1 d	19.86(39.86,76.04) *	2.14 ± 0.35*	2.05 ± 0.55*
2 d	16(34.5,61.75)*	2.124 ± 0.36*	1.94 ± 0.53*
3 d	13.25(32,63.75)*	2.09 ± 0.25*	1.75 ± 0.53*
1 wk	5.87(17.22,57.74)*	2.21 ± 0.28*	1.49 ± 0.51*
1 mo	11.2(20.24,59.41)*	2.18 ± 0.34*	1.32 ± 0.42*
3 mo	11.81(26.7,60.54)*	2.16 ± 0.24*	1.28 ± 0.43*
6 mo	18.04(42.57,58.21)*	2.12 ± 0.26*	1.44 ± 0.49*
12 mo	22.57(36.75,59.62)*	2.25 ± 0.16*	1.26 ± 0.22*

iPTH : intact parathyroid hormone; /: not monitored; iPTH levels are expressed as the median (interquartile range);Serum calcium and serum phosphorus are expressed as the mean ± standard deviation; A probability value of *p < 0.05 was considered to be statistically significant compared with those before the operation.

### Comparison of the effects of TPTX + AT and SPTX on laboratory indicators

**Fig. 2:**
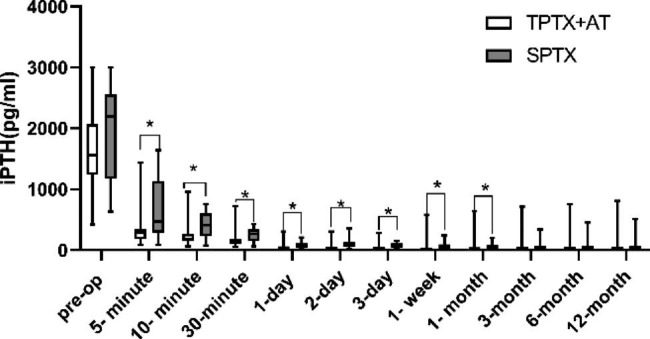
Comparisons of iPTH levels between TPTX + AT and SPTX

**Fig. 3:**
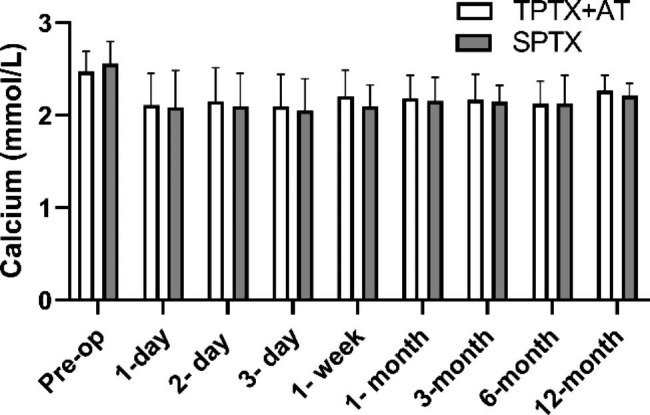
Comparisons of serum calcium levels between TPTX + AT and SPTX

**Fig. 4:**
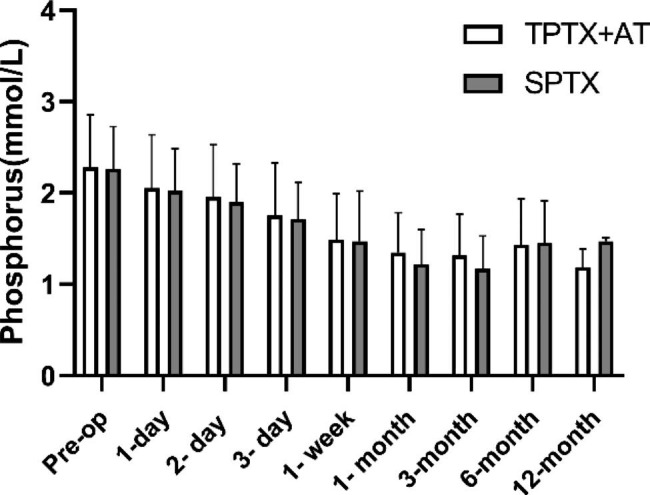
Comparisons of serum phosphorus levels between TPTX + AT and SPT

The follow-up data of iPTH, and blood phosphorus levels were significantly lower at each postoperative period than pre-op in both groups shown in Fig. 2. There were differences in iPTH between the two groups in the short term (within 1 month after surgery, *p value < 0.05), but no significant differences were seen in the long term (3 months after surgery). There was no significant difference in calcium and phosphorus values between the two groups during the follow-up period (Figs. 3 and 4).

### Improvement of symptoms after surgical treatment

One week after the operation, patients showed significant improvement in restless leg syndrome, pruritus, bone pain, and arthralgia (*P* < 0.05). However, improvements in insomnia were not observed until three months after surgery. At the six-month and twelve-month follow-up assessments, patients showed a significant improvement in all symptoms, including restless leg syndrome, pruritus, bone pain, arthralgia, and insomnia(*P* < 0.05), when compared with the preoperative period (Table 4).

**Table 4:** Analysis of VAS scores on symptoms before and after PTX.

VAS scores	Restless legs syndrome	*p*-value	Pruritus	*p*-value	Bone pain	*p*-value	Joint pain	*p*-value	Insomnia	*p*-value
Pre-op	7.19 ± 1.41		7.36 ± 1.82		7.06 ± 1.69		6.99 ± 1.41		5.81 ± 2.06	
1 wk	4.03 ± 1.11	*P* < 0.001	1.65 ± 0.97	*P* < 0.001	2.55 ± 1.02	*P* < 0.001	2.7 ± 1.07	*P* < 0.001	5.60 ± 1.93	*P* = 0.61
3 mo	2.19 ± 0.96	*P* < 0.001	1.33 ± 0.95	*P* < 0.001	2.04 ± 1.11	*P* < 0.001	1.33 ± 0.69	*P* < 0.001	4.18 ± 1.57	*P* < 0.001
6 mo	1.29 ± 0.9	*P* < 0.001	1.29 ± 0.94	*P* < 0.001	1.83 ± 0.95	*P* < 0.001	1.28 ± 0.59	*P* < 0.001	3.5 ± 1.24	*P* < 0.001
12 mo	1.21 ± 0.98	*P* < 0.001	1.14 ± 1.02	*P* < 0.001	1.45 ± 0.69	*P* < 0.001	1.39 ± 0.56	*P* < 0.001	2.71 ± 1.21	*P* < 0.001

Data are presented as the means ± SD. wk, week; mo, month;

### Improvement of quality of life after surgical treatment

Comparison of QOL scores at different periods (Table 5): The results showed that the quality-of-life scores of the eight dimensions of PTX patients were significantly higher than those before the operation at 6 months and 12 months after the operation (*P* < 0.05). The PCS score increased to 82.24 (6.59) points at 6 months after the operation and 86.98 (5.37) points at 12 months after the operation. The MCS score increased to 77.94 (8.27) points at 6 months after the operation and 82.23 (7.28) points at 12 months after the operation.

**Table 5:** Analysis of SF-36 scores on quality of life in different periods

SF-36 dimension		Evaluation time		
		Pre-op, m (sd)	6 mo, m (sd)	12 mo, m (sd)
Physical functioning		31.06 ± 13.89	83.94 ± 10.66*	89.31 ± 8.67*
Role-functioning physical		21 ± 24.56	91.94 ± 11.62*	94.03 ± 9.51*
Bodily pain		28.2 ± 8.75	90.85 ± 11.51*	93.55 ± 9.06*
General health		50.84 ± 11.18	62.24 ± 10.32*	71.03 ± 10.69*
Vitality		42.2 ± 13.17	64.9 ± 13.76*	69.79 ± 13.24*
Social functioning		64.04 ± 23.67	88.91 ± 9.9*	91.3 ± 9.46*
Role-functioning emotional		59.23 ± 33.73	77.7 ± 22.17*	88.2 ± 17.28*
Mental health		54.73 ± 19.3	75.38 ± 11.48*	84.5 ± 9.75*
Physical component summary score		32.78 ± 8.86	82.24 ± 6.59*	86.98 ± 5.37*
Mental component summary score		55.05 ± 13.38	77.94 ± 8.27*	82.23 ± 7.28*

Date are presented as mean ± SD; A probability value of * *P* ≤ 0.05was considered to be statistically significant compared with those before the operation ; wk, week; mo, month;

### Postoperative complications and recurrence

Regarding postoperative complications, hypocalcemia (35%) and hyperkalemia (22.5%) were relatively common (Table 6). Hypocalcemia had the highest incidence, with symptoms such as twitching at the corners of the mouth or numbness of the limbs resulting from a decrease in ionized calcium in the blood. However, even at a level as low as 1.24 mmol/L, no severe convulsions were observed, and most patients showed significant improvement or disappearance of symptoms after receiving sufficient intravenous and oral calcium supplementation. Although the incidence of hyperkalemia was also high, no patients developed malignant arrhythmias, and only 2 patients had atrial fibrillation, which converted to sinus rhythm after treatment. Postoperative bleeding was mostly due to minor bleeding at the surgical site or wound infection, without any cases of asphyxia or hypovolemic shock. Two cases of bleeding were from the gastrointestinal tract, unrelated to the surgery itself, possibly triggered by anticoagulant therapy and other gastrointestinal bleeding symptoms in patients with long-term dialysis. There were no patients with vocal cord paralysis and no patient died during the perioperative period. Three of the patients who underwent TPTX + AT experienced recurrence within 1 year, as did 2 of the patients who underwent SPTX. Of the patients in whom the blood iPTH levels did not drop by 50% within 5 min of complete excision of all parathyroid tissue, 3 patients did not achieve a 70% decrease within 10 min, and 9 patients did not achieve an 80% decrease within 30 min. One patient who had a recurrence within 1 year met the criteria for the 3 decreasing thresholds, and among the 10 patients who did not meet the criteria, 4 patients had a recurrence within one year (considered as long as any one of the time thresholds was not met).

**Table 6:** Postoperative complications and recurrence

Complication	TPTX + AT(n = 60)	SPTX(n = 20)	P value
Nerve injury	0(0%)	0(0%)	N/A
Hemorrhage	2(3.33%)	2(10%)	0.554
Convulsion	0(0%)	0(0%)	N/A
Cardiac arrhythmia	2(3.33%)	0	N/A
Incision infection	3(5%)	1(5%)	1
Hypocalcemia	23(38.33%)	5(25%)	0.279
Hyperkalemia	15(25%)	3(15%)	0.536
Hypoparathyroidism	1(1.67%)	0	N/A
Recurrence 1-year post-op	3(5%)	2(10%)	0.79

Data are expressed as n (%); Categorical data were analyzed with the Chi-square test. N/A: not applicable;

## Discussion

Our retrospective study collected data from 80 patients who underwent PTX, analyzing changes in hemoglobin (Hb), alkaline phosphatase (ALP), iPTH, serum calcium, serum phosphorus levels as well as health-related quality of life, plus clinical symptom improvement before and after surgery. The biggest highlight of this study is the comprehensive analysis of the effects of parathyroidectomy on hemodialysis patients with secondary hyperparathyroidism over a relatively long period of time (1 year) from both objective indicators (iPTH, serum calcium, and so on) and patients’ subjective perspectives (SF-36 score, VAS score). Additionally, we confirmed that the intraoperative fast parathormone assay was predictive of the recurrence of hyperparathyroidism.

We found that parathyroidectomy did not significantly affect hemoglobin levels in end-stage renal disease patients, possibly due to irreversible damage to the renal interstitium and a consequent decrease in erythropoietin production. ALP is a group of glycoproteins that exhibit phosphatase activity under alkaline conditions, catalyzing the hydrolysis of various phosphates. It is a reliable indicator of bone turnover, promoting bone mineralization and reflecting the overall bone metabolism status of patients [18. Our study demonstrated that postoperative ALP levels displayed a significant increase from 234.8(130.3,427.3) to 274.8(156.28,452.98) U/L during the first week after surgery, which is consistent with previous studies conducted by LEIKER et al. [19 reported a similar transient elevation of ALP levels after PTX surgery.

Given the short half-life of iPTH, which is only 2 to 4 min, a retrospective analysis found that the median half-life of iPTH during surgery was 3 min and 9 s [20. Therefore, there may be a rapid decrease in serum iPTH during the PTX period. On the other hand, the speed of iPTH decrease may indicate whether all parathyroid tissues have been removed. According to HIRAMITSU et al., serum iPTH decreased to 70% of preoperative levels within 10 min after surgery, indicating sufficient removal of parathyroid tissues [21. Serum iPTH should be tested more frequently at 5, 10, and 30 min after parathyroidectomy to detect missing or undiscovered parathyroid tissue during surgery. If the decrease in serum iPTH does not reach 70%, it is likely that not all parathyroid tissue has been removed. To exclude ectopic parathyroid tissues, adipose tissue near the thymus and carotid artery sheath should be removed. However, CONZO et al. suggested that immediate postoperative iPTH levels of 26.52 pmol/L can predict the success of surgery, even if the decrease rate is not less than 80% within 20 min [22. In this study, 5 patients had recurrence during the 1-year follow-up, and the recurrence may be due to undiscovered parathyroid tissues during surgery in some of these patients. Patients who did not meet the standard had a higher rate of recurrence in the follow-up period than those who met the standard. It was discovered that the intraoperative fast parathormone assay might predict the persistence and recurrence of hyperparathyroidism [12, 23]. We tracked iPTH levels during the SHPT procedure, and it also exhibited this manifestation.

In our study, hypocalcemia (35%) ranked the highest among postoperative outcomes. This could be related to the high incidence of postoperative hungry bone syndrome, where more free calcium enters the bone, resulting in hypocalcemia. Hypocalcemia was also found to be the most common complication in the study by ZHAO et al. (36.2%), especially in TPTX [24. Postoperative hypocalcemia has been reported to be a significant focus of subsequent treatment and care as it greatly affects the patient’s quality of life, readmission rates, and mortality [25. A meta-analysis showed that preoperative serum calcium, ALP) and iPTH levels were significant predictors of hypocalcemia in patients with SHPT after PTX [26. In addition, the total weight of parathyroid tissue removed during surgery has also been identified as a risk factor for postoperative hypocalcemia [24. No patient in our study had nerve damage, possibly because our operations were performed by the same team of well-trained surgeons who always exposed the recurrent laryngeal nerve and properly drained it after surgery, leading to a low incidence of postoperative issues. Patients receiving TPTX + AT treatment showed that their postoperative recurrence was easier to manage than those receiving SPTX because they did not need a second neck surgery with higher risks and complexity,but instead underwent surgery on a more practical forearm. In our study, one patient underwent recurrent forearm transplantation, but SPTX patients did not undergo this surgery due to the risks associated with neck surgery and the complexity of the second surgery. As Lau et al. described, the long-term probability of significant hypocalcemia with TPTX + AT was higher than that with SPTX [13. However, a few TPTX + AT patients may experience long-term serum iPTH values that are lower than normal (close to 0) due to transplant survival issues, and this requires low-temperature storage of the removed tissue during surgery. Unfortunately, our hospital lacks such equipment, so symptomatic calcium supplementation measures can only be taken to alleviate the impact on patients with persistently low iPTH after surgery.

Almost all patients experienced relief of symptoms such as skin itching, bone pain, and joint pain shortly after surgery. High concentrations of parathyroid hormone (PTH) can cause calcium and magnesium ions to accumulate on the skin surface and stimulate histamine release, leading to skin itching. PTH is a macromolecule that exists in low concentrations in the plasma and is poorly cleared by diffusion alone during hemodialysis, which can lead to PTH accumulation in the body over time and worsening skin itching in long-term dialysis patients. Moreover, the results of the SF-36 showed significant improvement in all aspects compared to preoperative patients, but the improvement in MCS was smaller than that in PCS 6 and 12 months after surgery. This may be because depression is the most common comorbid mental illness in end-stage renal disease patients, affecting an estimated 25% of all patients [27, which can have an impact on the assessment of MCS.

This study has several limitations that should be noted. First, it is a single-center retrospective analysis with a limited sample size, which may limit the generalizability of the findings. Second, it is important to recognize the potential limitations of evaluation tools, such as SF-36 and VAS scores, which are subject to individual subjectivity despite our effort to use uniform evaluation criteria. Additionally, it can be challenging to distinguish symptoms of SHPT from clinical symptoms of uremia, such as insomnia, depression, and bone pain, which may overlap and affect the interpretation of study results. Future studies with larger, multicenter samples and more objective symptom evaluation methods are warranted.

Hyperparathyroidism is a severe complication that significantly affects the quality of life and increases the risk of death in patients with end-stage renal disease due to inadequate renal supply. Our study findings indicate that regardless of the surgical method used, there were significant improvements in both quality of life and laboratory measures among patients. Several prospective investigations and Mata analyses have also supported these findings [28–31].

## Conclusion

In summary, we found that TPTX + AT and SPTX were both safe and effective treatments for SHPT in our SHPT patients, and that they both significantly improved the patients’ clinical symptoms, quality of life, and levels of calcium and phosphorus metabolism. The decrease in iPTH during the brief postoperative period was also within our expectations and may have had a positive effect on the patients’ rate of recurrence. However, we must acknowledge that the size of our sample is small and that patient perceptions play a role in how well patients are doing.

## Data Availability

The data that support the findings of this study are available on request from the corresponding author. The data are not publicly available due to privacy or ethical restrictions.
